# The Role of *cheA* Genes in Swarming and Swimming Motility of *Pseudomonas pseudoalcaligenes* KF707

**DOI:** 10.1264/jsme2.ME15164

**Published:** 2016-05-03

**Authors:** Stefano Fedi, Tania Triscari Barberi, Maria Rosaria Nappi, Federica Sandri, Sean Booth, Raymond J. Turner, Marcella Attimonelli, Martina Cappelletti, Davide Zannoni

**Affiliations:** 1 Department of Pharmacy and Biotechnology, University of BolognaVia Irnerio 42, 40126, BolognaItaly; 2 Department of Biological Sciences, University of Calgary2500 University Dr NW, Calgary, AB T2N 1N4, AlbertaCanada; 3 Department of Biosciences, Biotechnologies and Biopharmaceutics, University of Bari Aldo MoroPiazza Umberto I, 70121 BariItaly

**Keywords:** bacterial motility, chemotaxis genes, *Pseudomonas pseudoalcaligenes* KF707, swarming, swimming

## Abstract

A genome analysis of *Pseudomonas pseudoalcaligenes* KF707, a PCBs degrader and metal-resistant soil microorganism, revealed the presence of two novel gene clusters named *che*2 and *che*3, which were predicted to be involved in chemotaxis-like pathways, in addition to a *che1* gene cluster. We herein report that the histidine kinase coding genes, *cheA2* and *cheA3*, have no role in swimming or chemotaxis in *P. pseudoalcaligenes* KF707, in contrast to *cheA1*. However, the *cheA1* and *cheA2* genes were both necessary for cell swarming, whereas the *cheA3* gene product had a negative effect on the optimal swarming phenotype of KF707 cells.

Most microorganisms inhabiting heterogeneous environments are motile. Chemotactic behavior in bacteria is achieved by integrating signals received from receptors that sense the environment (1). Motile bacterial species appear to have retained a large number of genes involved in motility and chemotaxis during their “evolution”, and this genomic reservoir provides selective advantages and plays a significant role in the dynamics of microbial populations (13). Therefore, bacterial chemotaxis may be considered a prerequisite for population survival, metabolism, and interactions within ecological niches (13). It is also crucially involved in the colonization of plant roots by *Pseudomonas fluorescens* (3), the infection of plants by *P. syringae* (11) and *Ralstonia solanacearum* (25), and animal infections by *P. aeruginosa* (4). Chemotaxis is regarded as a selective advantage for bacteria that colonize contaminated environments and present chemotactic ability towards xenobiotic compounds (18).

The soil bacterium *P. pseudoalcaligenes* KF707, hereafter referred to as KF707, is known for its ability to degrade toxic pollutants such as biphenyls and polychlorinated biphenyls (PCBs) (5, 7), to which the strain is chemically attracted (21). However, toxic hydrophobic chemicals such as PCBs are often adsorbed to a non-aqueous-phase liquid and their low bioavailability is a limitation for the microbial remediation of contaminated sites (20). In this case, microbial contact with pollutants is facilitated by biofilm formation, in which chemotaxis plays a fundamental role (16), because swarming motility facilitates the spread of the biofilm (20). Recent findings have shown that KF707 *cheA1::Km* (previously named KF707 *cheA::Km*), a chemotactic mutant, exhibited impaired chemotaxis and biofilm development (22).

Bacterial species belonging to the genera *Pseudomonas*, *Vibrio*, and *Rhodobacter* have been shown to possess multiple gene clusters involved in chemotaxis-like signaling pathways, which may be involved in other cellular functions. *Myxococcus xanthus* contains up to eight chemotaxis-like pathways that play multiple roles such as spore-producing fruiting bodies and/or the production of extracellular polysaccharides (26). *P. aeruginosa* PAO1 possesses four operons, named *che*, *che2*, *pil-chp*, and *wsp* (12), some of which are involved in the control of cyclic-di-GMP production and biofilm formation (9).

The complete genome of strain KF707 was recently sequenced (accession number PRJNA187055) and annotated using RAST software (23). Sequence similarity searches were performed using BLAST software (http://www.ncbi.nlm.nih.gov/blast/blast.cgi) together with the conserved domain database (http://www.ncbi.nlm.nih.gov/cdd/), while multiple sequence alignments were performed with ClustalW software.

An analysis of the annotated genome allowed us to identify three gene clusters (named *che1*, *che2*, and *che3*) predicted to be involved in chemotaxis-like pathways (Fig. 1 and Table S1), two of which, *che2* and *che3*, were in addition to the *che1* gene cluster previously described by Tremaroli *et al.* (22). As multi-functional modular CheA proteins, the products encoded by *cheA2* and *cheA3* genes (CheA2 of 2528 aa and CheA3 of 588 aa) contain five domains (designated as P1–P5) (Fig. S1). CheA2 shows 72% similarity with the ChpA protein of *P. aeruginosa* PAO1. Furthermore, the arrangement of KF707 ORFs flanking the *cheA2* gene— coding for a CheB2 methylesterase, CheR2 methyltransferase, and PilGHIJ components—was similar to that found in the cluster *pil*-*chp* of *P. aeruginosa* PAO1 (Fig. 1) (24). In contrast to the CheA2 protein, CheA3 shows a conserved domain pattern similar to that described for CheA1. It possesses one P1 domain and lacks the signal receiver (REC or P2) domain (Fig. S1). The *che3* cluster includes ORFs coding for two methyl-accepting chemotaxis proteins (MCP), a chemotaxis regulator protein, histidine kinase CheA3-like, CheW3, and CheD3 proteins, CheR3, and CheB3. As shown in Fig. 1, the KF707 *che3* cluster had the same organization as the *che2* cluster of *P. aeruginosa* PAO1.

The novel finding in KF707 of the chemotaxis-like clusters *che2* and *che3* prompted us to verify their role in motility and chemotaxis. Swimming and plug chemotaxis assays were performed as described previously (19, 22), while swarming was assayed on swarm plates consisting of a swarming minimal medium supplemented with sucrose (0.5% [w/v]) and solidified with Bacto-agar (0.7% [w/v]). In order to test the specific role of *cheA* genes in motility and chemotaxis, the single-deletion mutants *ΔcheA2* and *ΔcheA3* and double-deletion mutants *cheA1::KmΔcheA2*, *cheA1::KmΔcheA3*, and *ΔcheA2ΔcheA3* were constructed using the conjugative plasmid pG19II (15). Either a *cheA2* or *cheA3* deletion was performed in the *cheA1::Km* mutant previously constructed to make *cheA1::KmΔcheA2* and *cheA1::KmΔcheA3*, respectively (22). A list of all strains and plasmids used in this study is provided in Table S2, and the primer pairs used for the construction of recombinant sequences and details on the procedure used to obtain the deletion mutants are shown in Table S3.

The results of chemotactic and motility tests are summarized in Table 1 and shown in Fig. S2. Among the three *cheA* genes identified in the KF707 genome, only *cheA1* appeared to be involved in chemotaxis and flagellum-driven motility (swimming motility) because *ΔcheA2*, *ΔcheA3*, and *ΔcheA2ΔcheA3* maintained the swimming phenotype and the capacity to move toward chemical attractants such as succinate, benzoate, and biphenyl (Table 1, Fig. S2).

Swarming is defined as the capacity of bacterial cells to spread over the agar surface in a social motile behavior (10). Cells move by means of flagellar rotation either by pulling with type IV pili or by producing slime and/or surfactants that facilitate surface hydration and lubrication. As shown in Fig. 2A and B and Fig. S2, *cheA1::Km*, *ΔcheA2* along with *cheA1::KmΔcheA2* and *ΔcheA2ΔcheA3* had a significantly smaller swarming area than KF707 W.T. cells. Notably, the lack of the *cheA3* gene did not repress swimming or swarming motility because *ΔcheA3* was swimming and swarming positive. These results indicated that the deletion of only the *cheA1* and *cheA2* genes negatively influenced swarming motility. The introduction of a *cheA3* deletion into *cheA1::Km* and *ΔcheA2* led to the swarming area of *cheA1::KmΔcheA3* and *ΔcheA2ΔcheA3* being significantly larger than those of *cheA1::Km* and *ΔcheA2* (Fig. 2A, B and Fig. S2). Conversely, the increase observed in the swarming area was not significant with *ΔcheA3* (Fig. 2A and B). The deletion of only *cheA3* may not be sufficient to induce a significant increase in the swarming area because of the interactions among CheA proteins and the interference of other unknown factors such as cyclic di-GMP, c-AMP, and quorum sensing signals (14, 16).

In order to complement the swimming and swarming motility phenotype, the *cheA1*, *cheA2*, and *cheA3* genes were expressed *in trans* using pSEVA cloning vectors (Table S3).

The *cheA1* gene product complemented swimming motility in all the mutants carrying *cheA1::Km* (*cheA1::Km*, *cheA1::KmΔcheA2*, and *cheA1::KmΔcheA3*) while swarming motility was partially complemented when the *cheA1* gene product was introduced in *cheA1::Km*, *cheA1::KmΔcheA2*, and *cheA1::KmΔcheA3*. Furthermore, the *cheA2* gene product partially complemented the swarming phenotype in *ΔcheA2*, *cheA1::KmΔcheA2* and *ΔcheA2ΔcheA3* (Fig. S3). Following the introduction of the *cheA3* gene in *cheA1::KmΔcheA3* and *ΔcheA2ΔcheA3*, we observed the recovery of the swarming phenotypes shown by *cheA1::Km* and *ΔcheA2* (Fig. S3). Previous studies demonstrated that swarming motility was affected by a hierarchical cascade of second messengers including cyclic di-GMP, c-AMP, biosurfactants, and quorum sensing signals (12, 14). The swarming motility of *P. aeruginosa* was also recently found to be inhibited by naphthalene degradation intermediates such as 1-naphthnol (17). Notably, 1-naphthnol also up-regulated the expression of *mexAB* genes coding for an efflux pump involved in antibiotic resistance (17). Overall, these findings indicate that while *cheA1* and *cheA2* genes play a specific role in the motility behavior of KF707, the *cheA3* gene product, although not directly involved in motility, may interact with the *cheA1* and *cheA2* products negatively affecting swarming.

As revealed by the present bioinformatic analysis, novel *che2* and *che*3 clusters are homologous to *P. aeruginosa* PAO1 *pil-chp* and *che2* clusters, respectively, which were reported to control PAO1 swarming motility (Fig. 1) (2, 24). Previous studies have shown that PAO1 ChpA acts as a histidine kinase that regulates the two response regulators, PilG and PilH using a phosphorylation mechanism. These two CheY-like response regulators lead to the production of cAMP mediated by cAMP cyclase activity. In turn, cAMP regulates swarming motility through a regulatory signal cascade (14). In KF707, the *che2* cluster codes for proteins homologous to Pil-Chp cluster products (Fig. 1). According to the evidence found in PAO1, *che2* cluster products represent the putative core of the signaling components in KF707. On the other hand, in *P. aeruginosa* PAO1, the Che2 proteins, homologous to KF707 *che3* cluster products, interact with the bacterial Che system under stationary growth conditions (6). Although a precise role for these proteins in the chemotaxis of PAO1 has not yet been established, Che2 proteins disrupted chemotaxis in *E. coli* K-12 when they were heterologously expressed. These findings suggest the interference of Che2 proteins with endogenous *E. coli* chemotaxis protein activities (6, 8). In KF707, the alteration observed in swarming motility with the *cheA3* deletion in a *cheA1* and *cheA2* minus background indicated that CheA3 protein activity negatively affects the *cheA1* and *cheA2* swarming phenotypes. On the basis of these results and according to the findings obtained for PAO1, CheA3 activity modulates the swarming motility of KF707. In contrast, neither CheA2 nor CheA3 influence swimming, which appears to be solely dependent on the CheA1 histidine kinase (a summary of the results on swimming, swarming, and chemotaxis with the strains KF707 and PAO1 are reported in Table S4).

In summary, these results draw attention to the motility of *P. pseudoalcaligenes* KF707 and provide new insights into the role of different *cheA* genes in swimming and swarming. Once the roles of *cheA* genes in the swarming and swimming motility of strain KF707 have been assessed (this work), further studies will be required in order to investigate how *che* gene clusters affect biofilm construction along with their expression under different growth conditions.

## Supplementary Material



## Figures and Tables

**Fig. 1:**
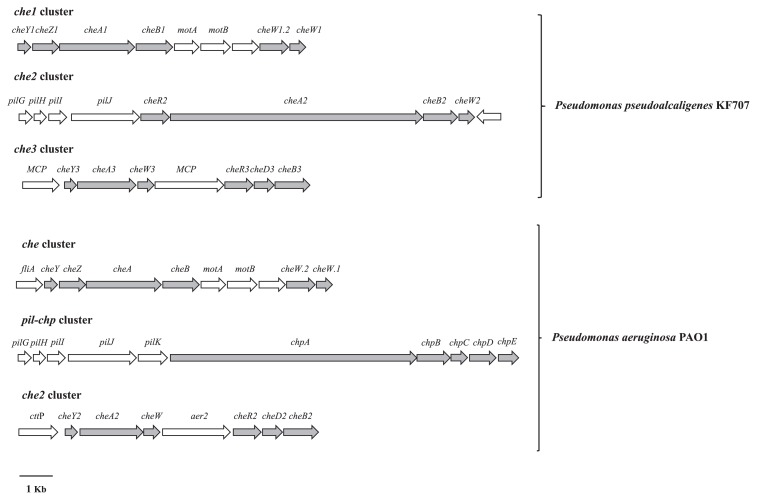
Genetic organization of three chemotactic systems of KF707 (*che1*, *che2*, and *che3* gene clusters, shown in grey) relative to those of *P. aeruginosa* PAO1.

**Fig. 2:**
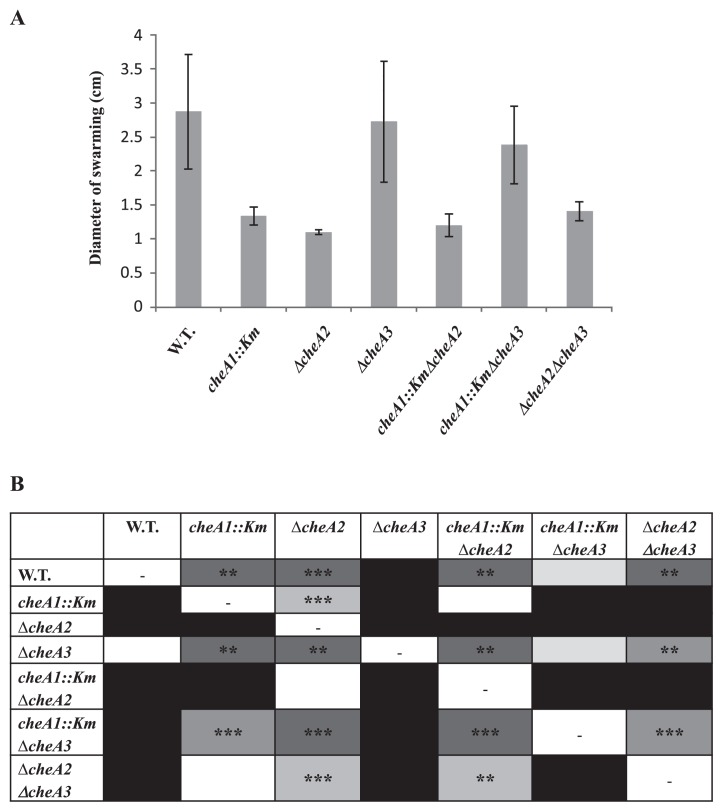
Swarming abilities of KF707 W.T. and *cheA* mutant strains. A) The diameters of swarming areas and the deviation standards measured after 7 d are reported (growth on solid sucrose swarming medium [0.7% agar] at 30°C) (plate diameter of 8.8 cm). B) A reduction in the swarming diameter (s.d.) is expressed as (s.d. of each strain in the row/s.d. of each strain in the column) ×100. The colors represent four ranges of s.d. reduction percentages. White, 0–25%; light grey, 25–50%; medium grey, 50–75%; dark grey, 75–100%. The black color represents a percentage value indicating an increase in s.d. instead of a reduction. A one-way ANOVA was performed to test the null hypothesis that there were no significant differences in the mean of s.d. of the seven strains, followed by Tukey’s post-hoc test. The results obtained were verified by performing a two-sample *t*-test within pairs of strains. ^**^P<0.01; ^***^P<0.001. Results reflect five experimental replicates for each strain.

**Table 1:** Specific roles of *cheA* genes (*cheA1*, *cheA2*, and *cheA3*) in swimming, swarming, and chemotaxis of KF707 W.T. and mutants.

KF707 strains	Motility^a^		Chemotaxis^a^		
	
	Swimming	Swarming	Succinate	Benzoate	Biphenyl
W.T.	+	+	+	+	+
*cheA1::Km*	−	−	−	−	−
*ΔcheA2*	+	−	+	+	+
*ΔcheA3*	+	+	+	+	+
*cheA1::KmΔcheA2*	−	−	−	−	−
*cheA1::KmΔcheA3*	−	+	−	−	−
*ΔcheA2ΔcheA3*	+	−	+	+	+

a Assay conditions as described in the text and Supplementary Materials.

Symbols and abbreviations used: +, present; −, impaired.
