# Supplementary material for: The Role of cheA Genes in Swarming and Swimming Motility of Pseudomonas pseudoalcaligenes KF707
Source: Microbes Environ. 2016 May 3;31(2):169–72. doi: 10.1264/jsme2.ME15164 (PMC4912153; doi:10.1264/jsme2.ME15164)
Supplement: Supplementary file 1 [file 31_169_s1.pdf]

Table S1 The KF707 *che* clusters genes

Gene	Gene product (aa)	Accession #	aa % identity	Organism	Accession #
***che1* cluster**					
cheY1	128	WP_003087110	99%	*P. aeruginosa sp.*	WP_031687269
cheZ1	262	WP_003457022	90%	*P. resinovorans* sp.	WP_028626566
cheA1	753	WP_003457024	95%	*P. resinovorans* sp.	WP_028626567
cheB1	367	WP_003457027	92%	*P. resinovorans* sp.	WP_028626568
motA	246	WP_003457029	93%	*P. resinovorans* sp.	WP_028626569
motB	297	WP_003457031	80%	*P. resinovorans* NBRC 106553	WP_016493842
cheW1.2	285	WP_003457036	85%	*P. resinovorans* sp.	WP_028626572
cheW1	159	WP_003457037	99%	*P. resinovorans* NBRC 106553	WP_028626572
***che2* cluster**					
pilG	135	WP_003455569	99%	*P. alcaligenes* sp.	WP_021218373
pilH	121	WP_003455567	98%	*P. resinovorans* NBRC 106553	WP_016490286
pilI	178	WP_003455565	92%	*P. resinovorans* NBRC 106553	WP_003455565
pilJ	682	WP_003455563	97%	*P. resinovorans* sp.	WP_028629554
cheR2	286	WP_003455560	88%	*P. resinovorans* sp.	WP_028629555
cheA2	2528	WP_003455558	81%	*P. resinovorans* sp.	WP_028629556
cheB2	343	WP_003455557	89%	*P. resinovorans* NBRC 106553	WP_016490291
cheW2	156	WP_003455555	85%	*P. resinovorans* sp.	WP_028629558
***che3* cluster**					
cheY3	121	WP_003450384	97%	*P. resinovorans* NBRC 106553	WP_016491325
cheA3	588	WP_003450382	87%	*P. resinovorans* NBRC 106553	WP_016491324
cheW3	161	WP_003450381	96%	*P. resinovorans* sp.	WP_028628133
aer	668	WP_003450379	88%	*P. resinovorans* sp.	WP_028628134
cheR3	281	WP_003450378	81%	*P. resinovorans* sp.	WP_028628135
cheD3	203	WP_003450377	89%	*P. resinovorans* NBRC 106553	WP_016491320
cheB3	349	WP_003450376	92%	*P. resinovorans* NBRC 106553	WP_028628137

Table S2 Bacterial strains and plasmids used in this study

**Strains**	**Relevant characteristics**	**Reference**
Pseudomonas pseudoalcaligenes		
W.T.	Wild Type	(7)
cheA1::Km[*]	*cheA::Km*, Km^R	(22)
ΔcheA2	*ΔcheA2*	This study
ΔcheA3	*ΔcheA3*	This study
cheA1::KmΔcheA2	*ΔcheA2*, *cheA1*::*Km*, Km^R	This study
cheA1::KmΔcheA3	*ΔcheA3*, *cheA1*::*Km*, Km^R	This study
ΔcheA2ΔcheA3	*ΔcheA2*, *ΔcheA3*	This study
Escherichia coli		
HB101	*rec*A *thi pro leu hsd*R, Sm^R	Boyer and Roulland-Dussoix (1969)
DH5α	*supE*44*hsdR*17*recA*1*endA*1 *gyrA*96*thi*1 *relA*1	Hanahan (1983)
Top10F'	F′{*lac*Iq, Tn*10*(TetR)} *mcr*A Δ(*mrr*-*hsd*RMS-*mcr*BC) Φ80*lac*ZΔM15Δ*lac*X74 *rec*A1 *ara*D139 Δ(*ara leu*) 7697 *gal*U *gal*K *rps*L (StrR) *end*A1 *nup*G	Invitrogen™
**Plasmids**		
pUC19	Amp^R, cloning vector	Sambrook et al. (1989)
pUC19*ΔcheA2*	Amp^R, carrying *cheA2* deleted fragment	This study
pUC19*ΔcheA3*	Amp^R, carrying *cheA3* deleted fragment	This study
pG19II	Gm^R, *sacB*, *lacZ*, cloning vector conjugative plasmid	(15)
pG19II*ΔcheA2*	Gm^R, *sacB*, *lacZ*, carrying *cheA2* deleted fragment	This study
pG19II*ΔcheA3*	Gm^R, *sacB*, *lacZ*, carrying *cheA3* deleted fragment	This study
pSEVA342	Cm^R, pRO1600/ColE1, *lacZ*α-pUC19	Silva-Rocha et al. (2013)
pSEVA532	Tc^R, pBBR1, *lacZ*α-pUC19	Silva-Rocha et al. (2013)
pSEVA342*cheA1*	Cm^R, pSEVA342 with *cheA1* gene in the Multiple Cloning Site (MCS)	This study
pSEVA532*cheA1*	Tc^R, pSEVA532 with *cheA1* gene in the MCS	This study
pSEVA342*cheA2*	Cm^R, pSEVA342 with *cheA2* gene in the MCS	This study
pSEVA532*cheA3*	Tc^R, pSEVA532 with *cheA3* gene in the MCS	This study

[*]Previously named *cheA::Km* in Tremaroli et al. (22).

Table S3 Primers used in this work

Primer list		
Deletion primers[a]	cheA2UP-fw	GCCGAAGCTTTGTTGTCCCTG
	cheA2UP-rev-ov	CTATAAGTGTAGCATGGTTGTCCGGG
	cheA2DOWNfw-ov	ATGCTACACTTATAGGACCGTGCGC
	cheA2DOWNrev	AAGCGGATCCGATGTCTTCTCTGTC
	cheA3UP-fw	GCCGAAGCTTTACCACCTGGTGTT
	cheA3UP-rev-ov	CTATAAGTGTAGCATTGGGTCACGGCG
	cheA3DOWN-fw-ov	ATGCTACACTTATAGTGATCCGGGTCCG
	cheA3DOWN-rev	AAGCGGATCCACGATGGGAACGATG
Cloning primers[b]	cheA1F	GCGAGAAGCTTCGACGCCGATGAAGAA
	cheA1R	ATAGTGGATCCTCAGCCACGGCGCGCGTA
	cheA2F	AACAATCTAGATCATGTCCCGTTTGACCAG
	cheA2R	ATTATCCTGCAGGACTGATCGGCATGACTA
	cheA3F	TACGTAAGCTTGAATGACGGGATGAACCAGTTC
	cheA3R	GATCAAGGATCCTAGGGGAGTTCATGCAG

[a] The outer primers used for the construction of recombinant sequences with deletion in *cheA2* and *cheA3* genes were constructed on the upstream region (forward primer) and the downstream region (reverse primer) of each gene. The forward and reverse primers were designed with *Hin*dIII and *Bam*HI restriction sites (underlined), respectively. The *cheA2*UP-rev-ov/cheA3UP-rev-ov and *cheA2*DOWN-fw-ov/*cheA3*DOWN-fw-ov primers were designed with the addition of the overlapping sequences 5'-CTATAAGTGTAGCAT-3' and 5'-ATGCTACACTTATAG-3'(grey shadowed), respectively, in order to join the flanking region together by the SOEing method as described previously (15; Izumi et al., 2007). The resulting PCR products with the deletion in *cheA2* or *cheA3* genes were double digested and cloned in *Hin*dIII/*Bam*HI restriction sites of pUC19. Sequencing from the M13 element of pUC19 using the primers M13-forward and M13-reverse verified the correct sequence of the constructs. Subsequently, fragments were cloned into the conjugative plasmid pG19II (15) double digested with *Hin*dIII/*Bam*HI and transferred by tri-parental conjugation to KF707 wild type strain by means of the helper strain *E. coli* HB101 (pRK2013). KF707 transconjugants were selected for their resistance to Gm and sensitivity to sucrose. After growth on sucrose at high concentration (10 g/L) in order to stimulate a double cross-over, mutants were isolated for their capacity to grow again on sucrose and loss of Gm^r. The recombination event with the consequent deletion of the target sequence was confirmed by sequencing the products obtained by colony PCR.

[b] In order to complement swimming and swarming motility phenotype, the *cheA1*, *cheA2*, and *cheA3* genes were expressed *in trans* in the cloning vectors pSEVA (Silva-Rocha et al., 2013). The *cheA1*, *cheA2* and *cheA3* sequences were amplified using these cloning primers listed and first cloned in *E. coli* Top10F' competent cells using the TOPO TA cloning kit (Invitrogen). The *cheA1* and *cheA3* PCR products were digested with *Hin*dIII and *Bam*HI restriction enzymes and ligated into pSEVA342 and/or pSEVA532 plasmids, while *cheA2* PCR product was digested with *Xba*I and *Sbf*I and ligated into PSEVA342 digested with the same restriction enzymes.

Table S4 Comparison of swimming, swarming, and chemotaxis phenotypes[a] of *cheA* mutants of *P. pseudoalcaligenes* KF707 and *P. aeruginosa* PAO1[b, c]

Deleted genes	Swimming		Swarming		Chemotaxis	
	KF707	PAO1	KF707	PAO1	KF707	PAO1
cheA1 (*cheA*)	-	-	-	ND	-	-
cheA2 (*chpA*)	N	N	-	N	N	N
cheA3 (*cheA2*)	N	ND	+	N	N	N

[a] +, improving effect; -, impairing effect; N, no effect; ND, not determined

[b] The homologous *cheA* genes in *P. aeruginosa* PAO1 are between brackets

[c] The data on PAO1 motility derive from Barken et al. (2008) and Kato (2008).

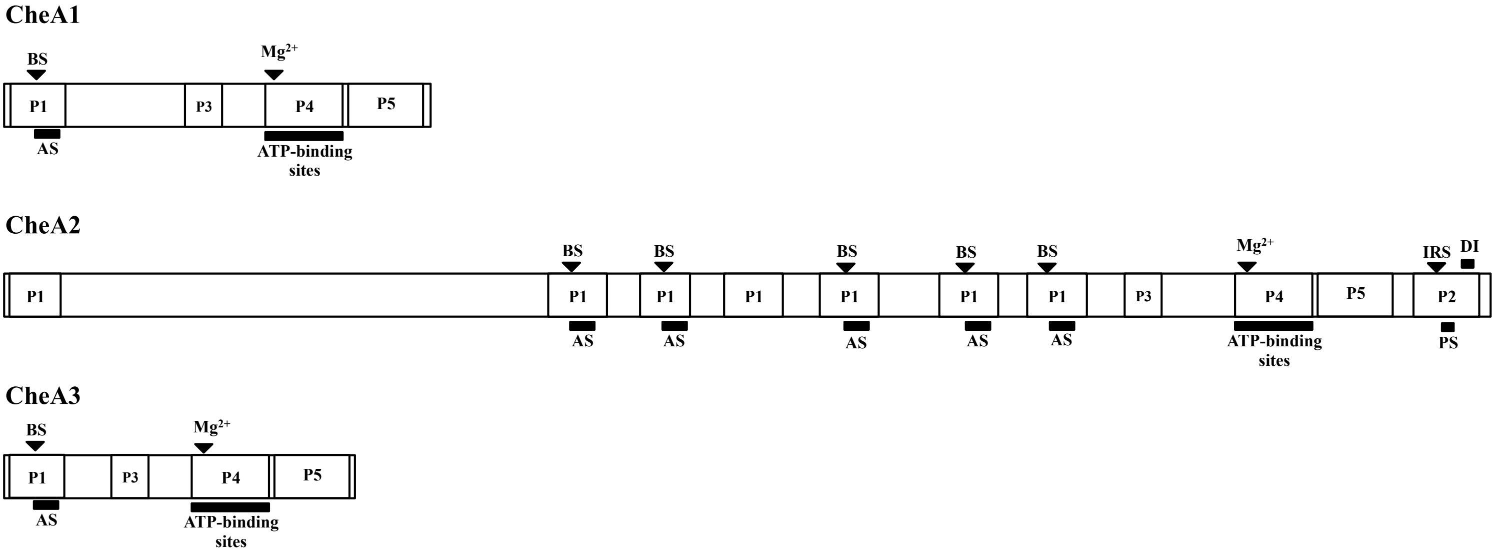

Figure S1 Schematic representation of the CheA histidine kinases (CheA1, CheA2 and CheA3) of KF707 based on amino acid sequence analysis. Relevant domains and features of the CheA proteins are indicated as follows: P1 domain = histidine-phosphotransfer domain; P2 domain = signal receiver domain; P3 domain = histidine kinase homodimeric domain; P4 domain = Histidine kinase-like ATPase domain; P5 domain = CheA regulatory domain; BS = putative binding surface; AS = putative active site; Mg^{2+} = Mg^{2+} binding site; DI = dimerization interface; IRS = intermolecular recognition site; PS = phosphorylation site. Active or binding sites composed by more than one conserved amino acid residue are shown as black rectangles while features constituted by one conserved residue are indicated by black triangles.

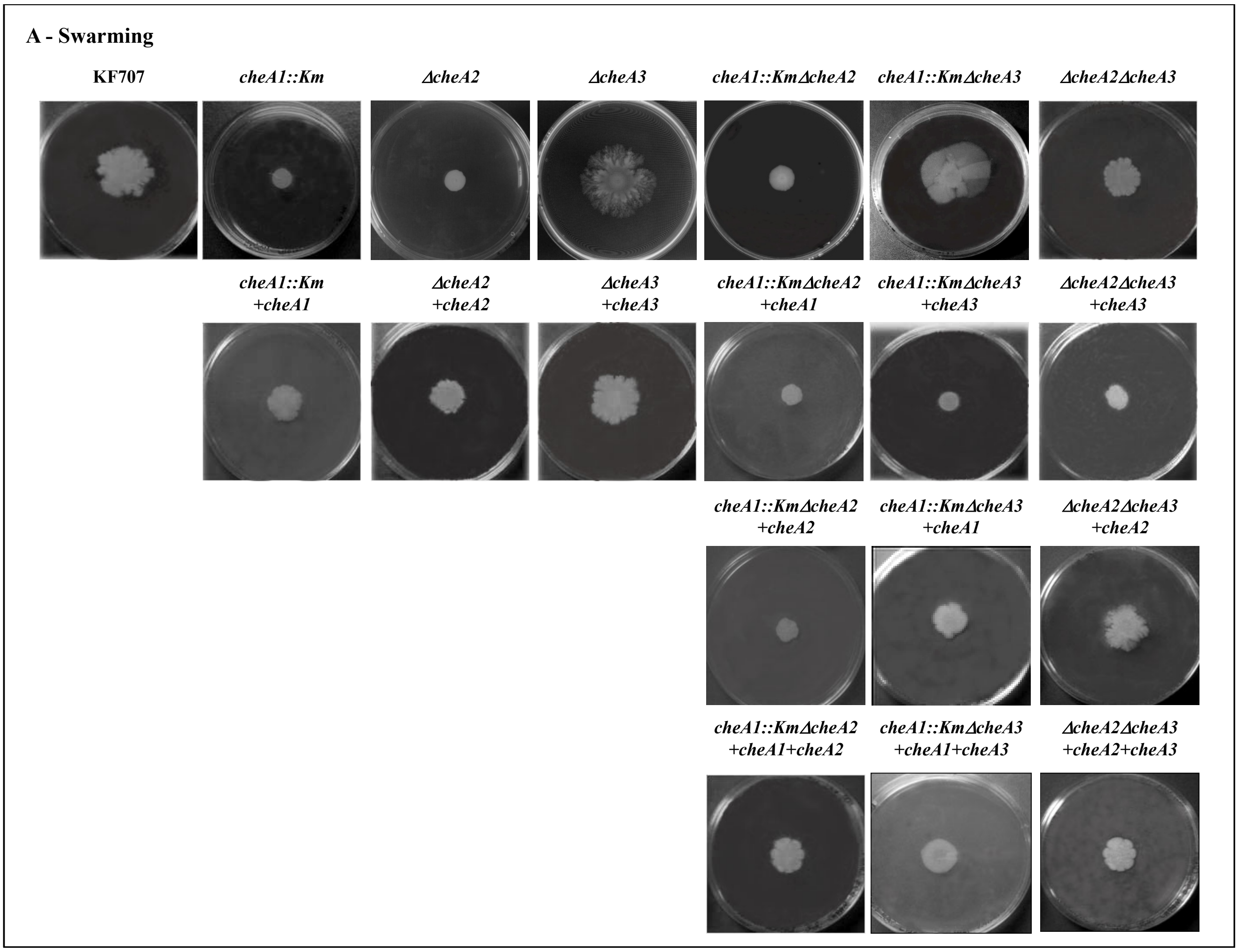

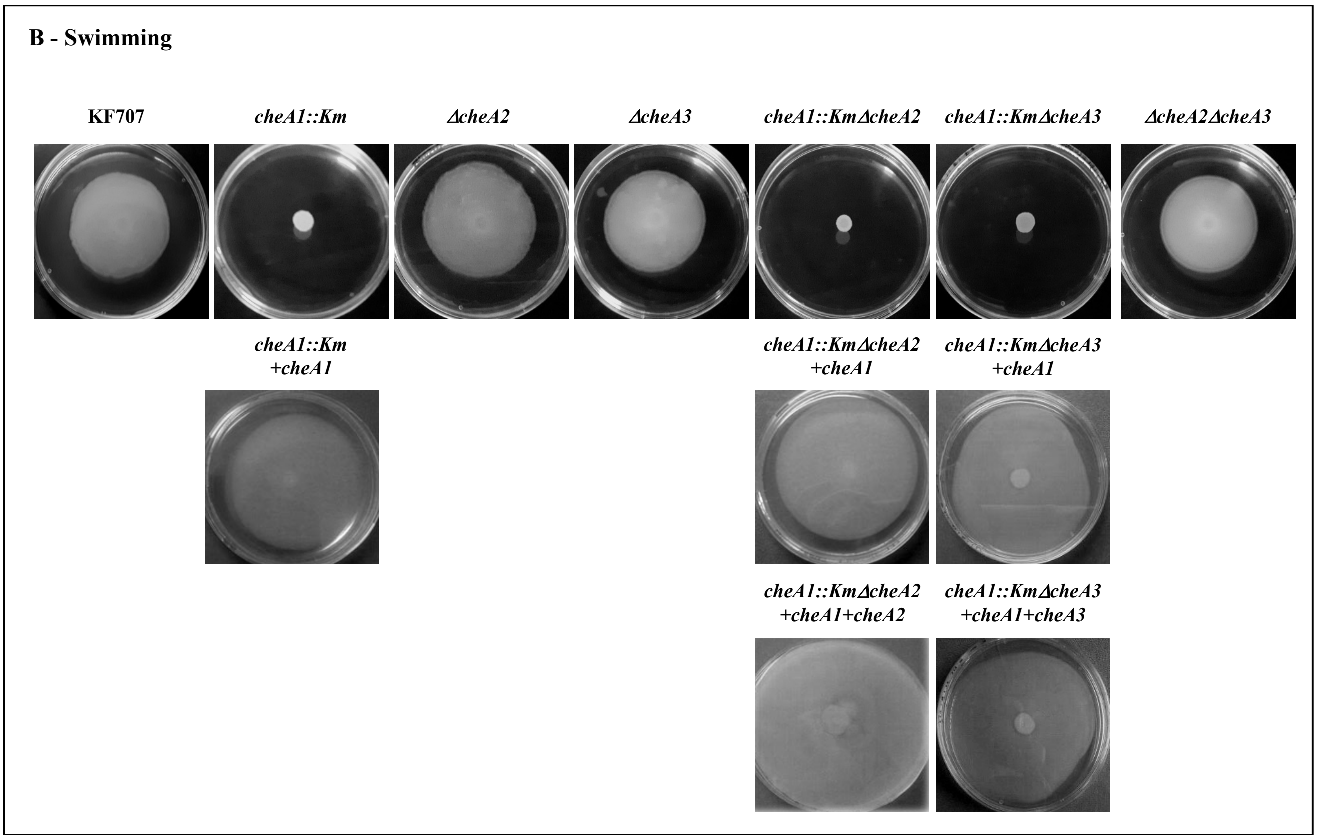

Figure S2 Swarming and swimming phenotype of KF707 W.T., single and double-deletion *cheA* mutants, single- and double-complementation mutants. Swarming areas were measured after 7 days while swimming haloes were determined after 24 hours (plates diameter of 8.8 cm) (see text for details).

Reference List of Supplementary Material

Barken, K.B., S.J. Pamp, L. Yang, et al. 2008. Roles of type IV pili, flagellum-mediated motility and extracellular DNA in the formation of mature multicellular structures in *Pseudomonas aeruginosa* biofilms. Environ. Microbiol. 10: 2331-2343.

Boyer, H.W., and D. Roulland-Dussoix. 1969. A complementation analysis of the restriction and modification of DNA in *Escherichia coli*. J. Mol. Biol. 41: 459-472.

Hanahan, D. 1983. Studies on transformation of *Escherichia coli* with plasmids. J. Mol. Biol. 166:557-580.

Izumi, K., M. Aramaki, T. Kimura, et al. 2007. Identification of a prosencephalic-specific enhancer of SALL1: comparative genomic approach using the chick embryo. Pediatr. Res. 61:660-665.

Kato, J. 2008. *Pseudomonas* motility and chemotaxis, p. 109-128. *In* B.H.A. Rem (ed.), Pseudomonas. Wiley-VCH, Weinheim

Sambrook, J., E.F. Fritsch, T. Maniatis. 1989. Molecular cloning: a laboratory manual. In: Sambrook J., E.F. Fritsch, T. Maniatis, (ed.s), 2nd edn. Cold Spring Harbor, N.Y. Cold Spring Harbor Laboratory Press.

Silva-Rocha, R., E. Martinez-Garcia, B. Calles, et al. 2013. The Standard European Vector Architecture (SEVA): a coherent platform for the analysis and deployment of complex prokariotic phenotypes. Nucleic Acids Res. 41:D666-D677.
